# Commercial Complementary Food in Germany: A 2020 Market Survey

**DOI:** 10.3390/nu14183762

**Published:** 2022-09-13

**Authors:** Ute Alexy, June Joann Dilger, Stefanie Koch

**Affiliations:** Department of Nutritional Epidemiology, Institute of Nutritional and Food Science, University of Bonn, 44225 Dortmund, Germany

**Keywords:** commercial complementary food, infants, toddlers, fortification, sugar, salt, beverages

## Abstract

As consumption of commercial complementary food (CCF) during infancy and toddlerhood is common, the aim of the present study was to describe the current (2020) German market of CCF products targeted at infants and toddlers with a special focus on ingredients, macronutrients, and the practice of nutrient fortification. Information on age declarations, ingredients, energy and nutrient contents, and nutrient fortification was obtained in a market survey by contacting the producers and searching manufacturers’ websites. Each product was assigned to 1 of 13 product categories (menus, milk–cereal–meal, fruit–cereal–meal, oil, vegetables, meat, fish, fruits, cereals, snack foods, pouches, desserts, beverages). Descriptive statistics were used. We identified 1057 CF products on the German market (infants’ CCF (<12 months): *n* = 829; toddlers’ CCF (>12 months): *n* = 228)). The highest protein content (% of energy content, %E) was found in meat products. In pouches, beverages, cereal fruit meals, and fruits, more than 50% of energy came from total sugar. The highest median salt content was found in toddlers’ menus and desserts. Around one-third of infants’ CCF products and one quarter of toddlers’ products were fortified with nutrients. Vitamin B1 (thiamin) was the most frequently fortified nutrient, followed by vitamin C, iron, calcium, and vitamin D. Apple was the type of fruit listed most often in products with fruits, whereas carrot was the most frequent vegetable among CCF with vegetables. In particular, the high total sugar content of most CCFs currently available on the German market may promote unhealthy dietary habits. Parents need to be advised about the optimal selection of products.

## 1. Introduction

Infants and young children toddlers are a special group in terms of nutrition. First, diet during this period has been found to be related to long-term health effects [1]. Additionally, infants’ taste preferences are shaped with long-lasting effects for later life [2,3,4]. Second, the motor skills that are just developing require a special consistency of food, which changes in the first year of life from liquid to pureed or soft foods to solid foods. Third, the high growth rate leads to a high energy requirement in relation to body weight [5,6]. A recommended macronutrient pattern is thus characterized by high fat content [7] (45–50% of energy from birth to 4 months, 35–45% from 4 to 12 months, and 30–40% from 1 to 3 years [6]), but protein intake should be limited [7,8]. There is no reference value for dietary fiber in infants; for toddlers, the same reference value is applied as that for older children and adults (14.6 g/1000 kcal) [6]. Potentially critical micronutrients in the early years of life include iron, vitamin D, and iodine, along with alpha-linolenic acid (ALA) and docosahexaenoic acid (DHA) [5].

In the first months after birth, breast milk is regarded as the normative standard for infant feeding. In Germany and Europe, exclusive breastfeeding is recommended for 4–6 months [7,9], whereas internationally exclusive breastfeeding is recommended for approximately 6 months [10,11]. Thereafter, infants should receive appropriate complementary foods along with continuous breastfeeding [7,9,11]. While breast milk provides enough energy and all the essential nutrients after birth, complementary foods are more variable and have to be combined to meet nutrient requirements [9,12].

Food-based dietary guidelines (FBDGs) for this age group can support parents and caretakers to select appropriate complementary foods. Such food pattern recommendations for infants and toddlers were developed recently by the US Dietary Guidelines Advisory Committee and provide an approximate amount of food groups for this age [13]. The official German FBDGs for infants go into more detail and provide meal-specific guidelines [14,15,16]. Developed for the first time by the former Research Institute of Child Nutrition, Dortmund (FKE), in the 1980s, the guidelines have been repeatedly modified but not fundamentally changed. The last relaunch with an adjustment to new reference values and some changes in the selection and quantities of foods was carried out in 2020 [14]. These FBDGs provide for three precisely defined meals in succession, that is, a vegetable–potato–meat meal, a milk–cereal–meal, and a cereal–fruit meal. In combination with breastfeeding or formula feeding and supplementation (vitamin D, fluoride), these three meals fulfill the reference values for the second half year of life [14,15]. For toddlers, the FBDGs in Germany are limited to general recommendations on food consumption (abundant plant foods, moderate animal source foods, sparingly sugar, sweet, and snack foods). In addition, it is pointed out that products marketed specifically for children are not necessary [17]. For infants, both the preparation of homemade complementary food and the use of commercial complementary food (CCF) are mentioned as possible options [9].

Homemade complementary food needs more time and effort but are less expensive, whereas CCF products are processed and convenient products. In addition, homemade complementary food has the potential to provide a greater variety of flavors and textures, whereas food variety in CCF is reported to be lower [18]. Some nutritional characteristics of CCF are required by law in the European Union. For example, maximum contents of sucrose or sugars were set, if these ingredients were added to a product. Furthermore, upper limits for sodium content were defined [19]. Nevertheless, market surveys repeatedly show that additions of salt and sugars in CCF are common in Europe and other countries [20,21,22,23]. Another point of concern is the low variety of ingredients in CCF [18,24]. Data from Germany [18], the US [25], and Canada [24] showed an association between a high consumption of CCF and low dietary variety, in particular with regard to fruit and vegetables [18,25]. Furthermore, CCF is in part fortified with nutrients, providing the opportunity for increasing nutrient intakes during infancy [26], in particular, in countries with a high prevalence of malnutrition.

Consumption of CCF during infancy and toddlerhood is common in Germany and Europe [20,25,27]. In the German DONALD study, only 20% of complementary food was homemade during infancy and toddlerhood, but 60% was CCF and the remaining 20% a combination of both [27]. In the more recent CHOP study, almost all infants consumed CCF up to the age of 9 months, and 1172 different products were reported in >1000 dietary records collected from 4- to 24-month-old infants from five European countries [20]. Due to the widespread use, data on the nutritional quality and composition of CCF are required.

The last market survey by CCF in Germany was conducted 20 years ago. At that time, there were more than 600 products on the market that had a high energy but low fat content. More than two-thirds of the products were fortified, mostly with vitamin C [28]. A more recent cross-sectional survey of manufacturer websites described the nutritional adequacy of commercial complementary cereals (*n* = 164 from 15 brands) on the German market [29]. In this study, a high total sugar content (14 ± 15 g/100 g) and low contents of zinc, iron, and iodine were demonstrated. Half of the products (52%) contained whole grain ingredients [29].

However, cereals only make up a small part of the market for CCFs. Hence, the aim of the present article was to describe the current market of CCF targeted at infants and toddlers in Germany. In detail, the following questions will be investigated: How many products from how many manufacturers are available, and do the product categories correspond to the German food-based dietary guidelines? Do the declared main nutrients correspond to the respective reference values for nutrient intake? Which nutrients are fortified, and is the fortification practice suitable to close gaps in potentially critical nutrients? What is the variability in ingredients, and do the products appear to promote healthy dietary behaviors? For this purpose, a survey of CCF for infants and young children in Germany was conducted in 2020, and the declared nutrients and ingredients were evaluated.

## 2. Materials and Methods

The survey started with the identification of relevant retailers, manufacturers, and brands in April 2020 through visits to supermarkets and discounters in Bonn and on websites. Afterwards, all identified producers were contacted to obtain declared product information (May to July 2020). In case of no response, the information was researched via the manufacturers’ websites.

Each product was assigned to one product category:

Full meals:

- Menus: declared as “baby menus” (for age group 5 to 7 months) and “junior menus” (for age group 8 to 12 months), composed of vegetables, starchy food (e.g., potatoes), some products with meat or fish, some products with added oil and/or fruit as ingredients, including soups and stews, offered in jars;
- Milk–cereal–meal: declared as “milk porridge“, “porridge“, “good evening porridge“, “good night porridge“, composed of milk and cereals, some products combined with fruit, offered as ready-to-eat meal in jars, bottles (drink meals), or instant products for the preparation with water;
- Fruit–cereal–meal: composed of fruit and cereals, often declared as “muesli“, offered in jars

Meal components:

- Oil: oils intended to prepare infant foods (“Beikostöl”);
- Vegetables: Pureed vegetables in jars, in part mixed with starchy food (e.g., potatoes or cereals), and oil
- Meat: pureed meat, in part with starchy foods and/or oil, offered in jars;
- Fish: pureed fish, in part with potatoes, offered in jars;
- Fruits: pureed fruits, in part mixed with dairy (e.g., yoghurt), cereals, or vegetables, offered in jars;
- Cereals: flakes or popped cereals, in part mixed with dried fruit pieces, offered as instant products

Snacks and beverages:

- Snack foods: biscuits, waffles, rusk, or bars;
- Pouches: pureed fruit partly mixed with vegetables, cereals, or dairy, offered in compressed plastic bags with a spout and a screw cap, from which the contents can be sucked out;
- Desserts: declared as desserts or pudding, offered in jars;
- Beverages: juices from fruit or vegetables, in part mixed with water or tea, teas, offered as instant products, tea bags, or ready-to-drink products in bottles

Full meals (i.e., menus, milk–cereal–meals, and cereal–fruit–meals) correspond to the above-mentioned meals within the German infants’ FBDGs [9,14]. The term full meals means that they are intended as a complete, nutritionally adequate meal, without the need to add other ingredients (except water in the case of instant milk–cereal meals).

Meal components (i.e., cereals, fruits, vegetables, meat, fish, and oil) can be used for the preparation of these meals. Some meal components can also feed on their own (e.g., fruits).

Snacks (i.e., pouches, snack foods, and desserts) do not correspond to FBDGs [14,15,16].

Data entry of the surveyed products was performed using Microsoft Excel (version 2016; Washington, America). The product characteristics included the brand and product name of the manufacturer, age declaration, declared ingredients, energy and nutrient contents, and nutrient fortification.

If sodium was declared instead of salt, the amount of salt was calculated. If nutrient contents were declared as “less than” (e.g., <20 mg sodium), the threshold value was used instead (20 mg sodium).

Products with added sweetening foods (i.e., sugar, syrup, honey, fruit concentrates, and fruit juice) according to the WHO definition of free sugar [30], salt (i.e., iodized salt, sea salt, table salt), and added fat/oil were identified according to the ingredient list.

Products were stratified into infants’ CCF (declaration < 12 months) and toddlers’ CCF (declaration “after 12 months”, “after 1 year”, or “1–3 years”).

Descriptive statistics were performed with SAS^®^ procedures (version 9.2; Cary, NC, USA) and included frequencies and percentages for categorical variables and median and quartiles for continuous variables.

As this study does not include humans or animals and refers only to freely available information, which is declared on the products, no ethical approval was necessary.

## 3. Results

### 3.1. CCF Products

Overall, 1057 CCF products of 27 brands were identified (1 to 210 products per brand), produced by 21 companies. Of these, 829 CCF products (78%) were declared for infants, and 228 (22%) products were declared for toddlers (Table 1).

Full meals made up more than one-third of infant CCF (39.6%, Table 1). Among full meals, more than half were menus (Table 1). Most menus were made with meat (*n* = 98, 54.5% of menus), followed by vegetarian menus (*n* = 50, 27.7%) and fish menus (*n* = 17, 9.4%). Fifteen menus (8.3%) were declared as “soups”.

Milk–cereal–meals were offered as instant products to be prepared with water (*n* = 49, 57.6% of milk–cereal–meals) or as ready–to–eat meals in jars (*n* = 30, 35.3%). Six products (7.1%) were offered as drink meals.

Cereal–fruit–meals accounted for around 20% of full meals (Table 1).

Among meal components, fruit products accounted for around half of the products (Table 1). The majority of fruit products were made from pure fruits, either single fruits (*n* = 20, 14.6% of fruit products) or a mix of fruit types (*n* = 89, 65.0%). Some fruit products were mixed with dairy (*n* = 25, 18.2% of fruit products) or vegetables (*n* = 3, 2.2%).

Among vegetable products, single vegetables (*n* = 21, 33.3%) or mixtures from different types of vegetables (*n* = 19, 30.2%) accounted for one-third of the products. Some vegetables were mixed with potatoes (*n* = 18, 28.6%) or fruits (*n* = 5, 7.9%).

Infant cereals accounted for around 10% of infants’ CCF products. Meat, oil, and fish were the meal components with the lowest number of products (Table 1).

The majority of snacks were pouches, followed by snack foods and desserts (Table 1).

Among pouches, three products were made only from vegetables (2.7%); the other pouches were prepared from fruits (*n* = 42, 37.8%) or mixtures of fruits with dairy (*n* = 9, 8.1%), vegetables (*n* = 22, 19.8%), or cereals (*n* = 35, 31.8%).

Of the beverages (Table 1), around two-thirds were teas or mixtures from juice and water or tea (*n* = 33, 61.1%); 21 products (38.9%) were pure juices.

The majority of infant CCF was declared for younger ages (“after 4 months”/“from the age of 5 months”); only 13% of infants’ CCF was declared to be “from the 10th to 12th months” (Table 1).

Snack foods (*n* = 77, 33.8%) were the predominant category among toddlers’ CCF, followed by pouches (*n* = 60, 26.3%), menus (*n* = 40, 17.5%), and beverages (*n* = 26, 11.4%). Six products (2.6%) were categorized as desserts. Toddlers’ cereals (referred to as mueslis, *n* = 12, 5.3%), noodles (*n* = 2, 0.9%), and sauces (*n* = 5, 2.2%) accounted for less than 10% of toddlers’ CCF. Of toddlers’ menus, 6 products (15.0%) were vegetarian menus, and 3 products were prepared with fish (7.5%).

### 3.2. Declared Energy and Nutrient Contents

Energy density (kcal/100 g) was highest in infants’ or toddlers’ snack foods. The highest protein content was found in cereals. The maximum fat content was found in toddlers’ snack foods and cereals. Saturated fatty acids exceeded 1.5 g/100 g only in meat products and in toddlers’ snack foods and desserts. Snack foods and cereals, for both infants and toddlers, had the highest carbohydrate contents (>60 g/100 g). Total sugar content was around 10 g/100 g in fruit–cereal–meals, fruits, and pouches and nearly 30 g/100 g in toddlers’ snack foods. Except toddlers’ menus and desserts, salt content was <0.9 g/100 g (Table 2).

**Figure 1:**
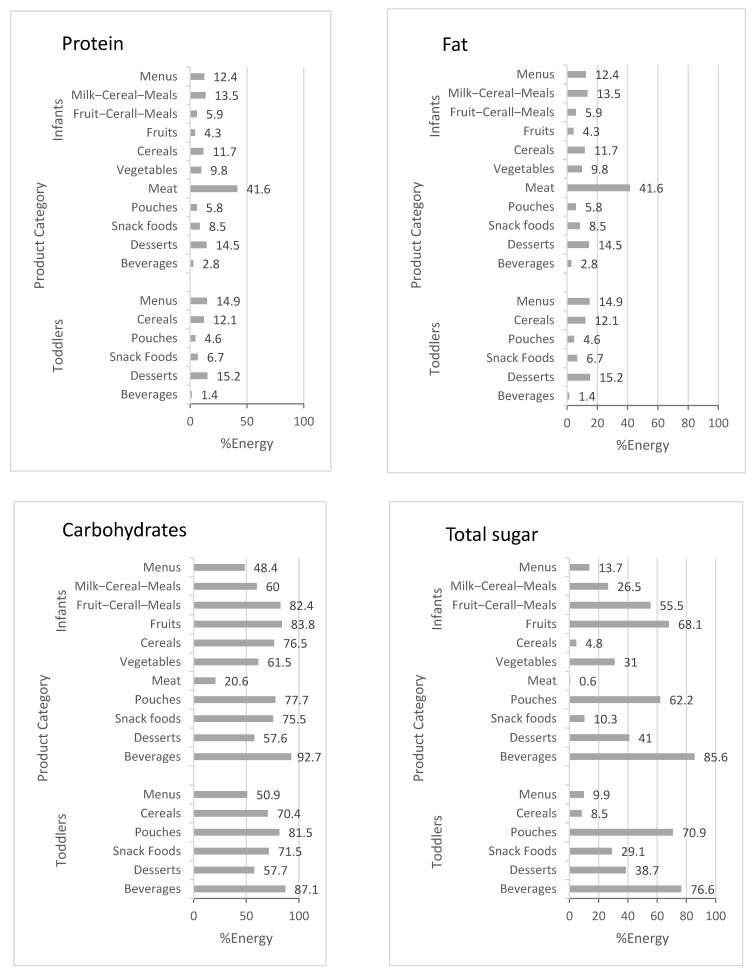
Declared macronutrient content (% of energy content) of commercial complementary food products (means of product category), results of a 2020 market survey in Germany.

Expressed as % of energy, the highest protein content was found in meat products, followed by menus for infants and toddlers and toddlers’ desserts. Carbohydrate content was lowest in meat (20.6%). In all other product groups, carbohydrates provided between around 50% and 90% of the energy content. In meat products, cereals, and toddlers’ menus, the contribution of sugar to energy content was less than 10%. More than 50 %E sugar was found in pouches, beverages, cereal fruit meals, and fruits (Figure 1).

### 3.3. Fortification

Around one-third of infants’ CCF products and one quarter of toddlers’ products were fortified with nutrients. The highest fortification prevalence among infants’ CCF was found in cereals, milk–cereal–menus, snack foods, and beverages. In toddlers’ CCF, cereals was the category with the highest prevalence of fortification, followed by snack foods and pouches. Vitamin B1 (thiamin) was the most frequently fortified nutrient, followed by vitamin C, iron, calcium, and vitamin D (Table 3).

CCFs with cereals (i.e., milk–cereal–meals, fruit–cereal–meals, and cereals) were the categories most often fortified with vitamin B1, products with fruits (i.e., fruits, beverages, pouches), and milk–cereal–meals with vitamin C. Infant menus and milk–cereal–meals were the categories most often fortified with iron. Milk–cereal–meals were also most often fortified with calcium and vitamin D, and the only category fortified with iodine (Table 3).

### 3.4. Ingredients

**Figure 2:**
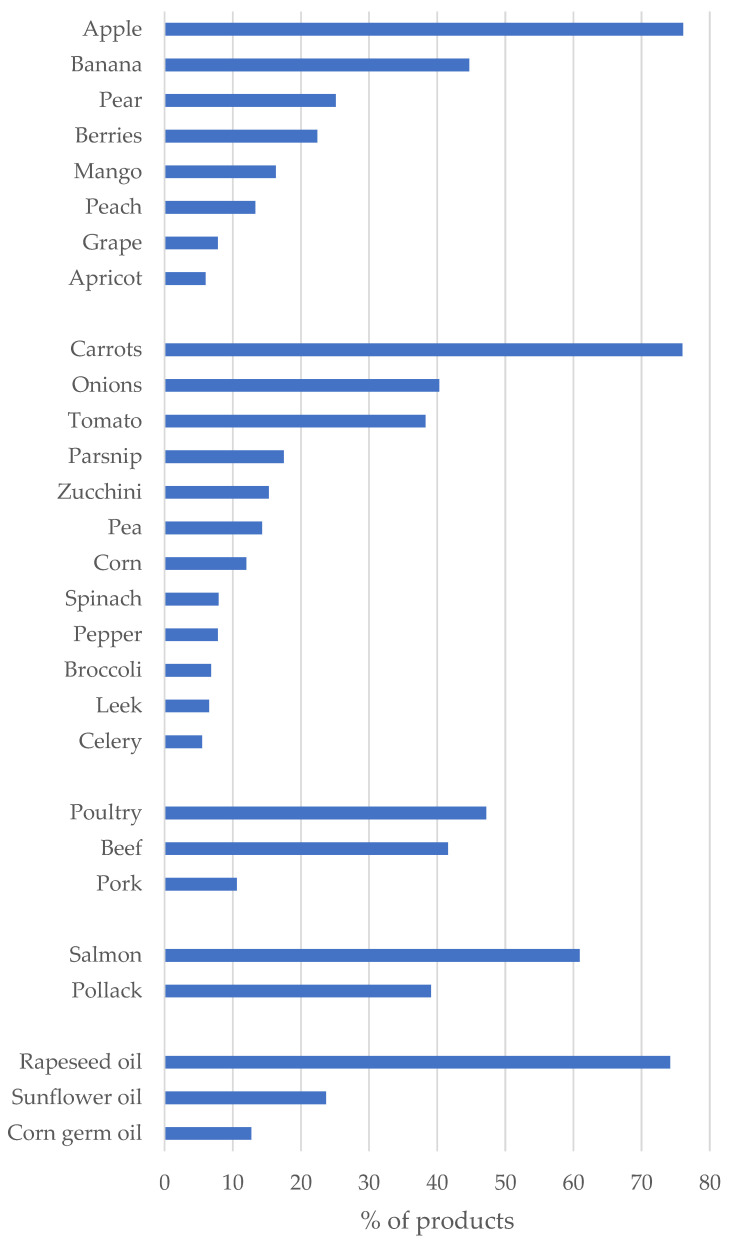
The most frequent ingredients (fruits, vegetable, meat, fish, and fat/oil, listed in at least ≥5% of products) in commercial complementary foods for infants and toddlers, results of a 2020 market survey in Germany (values are % of products containing fruits (i.e., fruit–cereal–meals, fruit products, fruit pouches, milk–cereal–meals in jars; *n* = 398), vegetables (i.e., menus, vegetables, and vegetable pouches; *n* = 308), meat (menus and meat products; *n* = 142), fish (fish menus and fish jars; *n* = 23), and added fat/oil (in menus and vegetable products; *n* = 283)).

Apple was the type of fruit listed most often in products with fruits, whereas carrot was the most frequent vegetable among CCFs with vegetables. Poultry (e.g., chicken, turkey) was the most frequent meat. Only two species were used for fish products (salmon and pollack). Four of five infants’ and toddlers’ menus and vegetables were prepared with added oil, predominately rapeseed oil (Figure 2). In 78 products (27.6%), a combination of rapeseed oil and other oils was used.

Sweeteners (i.e., sugar, honey, syrup, juice) were used in 233 products (23.8% of all CCF products, excluding beverages, *n* = 977). Juice was the sweetener used most often (*n* = 195, 83.7% of sweetened products), followed by sugar (*n* = 38, 16.3%), honey, and rice syrup (both *n* = 3, 1.3%). Other sweeteners (e.g., glucose, fructose, high-fructose corn syrup) were not found in CCF.

Among cereal-based products, whole grains were listed as ingredients in 139 products (61.0%).

Salt was added in *n* = 62 infants’ menus (34.4% of all menus), always as iodized salt. Four infants’ pouches were prepared with an oat mixture with added sea salt (3.6%). In toddlers’ menus, 80% (*n* = 32) of products were prepared with added iodized salt, two further products with sea salt (11.7%). Two toddlers’ snack foods were prepared with iodized salt and sea salt, respectively (each 2.4%).

Flavors, herbs or spices were used in 190 products (18.0% of all CCFs). Vanilla (*n* = 35, 18.4% of flavored products) was used in milk–cereal–meals (*n* = 16), toddlers’ CCF (*n* = 10), and desserts (*n* = 5). Seventeen products (8.9%) contained cacao, predominantly milk–cereal–meals and snack foods. Six products were flavored with cinnamon, and five beverages (teas) were flavored with aroma. Herbs (e.g., parsley, basil, oregano, marjoram, thyme, lovage, dill) were listed as ingredients in 58 infants’ or toddler’ menus, curry, turmeric, and/or ginger in seven products.

## 4. Discussion

### 4.1. Products

Compared with a similar study conducted 20 years ago in Germany [28], the number of infants’ CCFs has clearly increased (in 2020: infants’ CCFs from *n* = 598, toddlers’ CCFs from *n* = 38). A recent survey of infants’ CCFs in 10 European countries [31] included between *n* = 99 and *n* = 768 baby foods per country. These figures are also below the products identified in this market survey.

The declared age categories (after 4th month/from the age of 5 months onwards) fit with the current German recommendations on the timing of the introduction of complementary food [9]. However, there were also milk–cereal–meals or fruit–cereal–meals declared for early ages, although according to the German FBDGs [14], the introduction of these meals should start later on.

The vast majority of products (i.e., full meals and meal components) fit the meal recommendations of the German infant FBDGs. Menus accounted for the largest share of full meals. The majority of menus were produced with meat. As iron requirement during the complementary feeding period is high, a meat meal is recommended as first complementary food in Germany, whereas, internationally, iron-fortified cereals are recommended [7,12,14,32]. It is worth mentioning that in Germany, only one iron-fortified infant cereal was available. However, it should be noted that common whole grains (e.g., oat, millet, or wheat) have a high natural iron content, but bioavailability is lower than that of iron from meat [32].

In Germany, in contrast to other countries [33,34], fish has hardly been included in complementary feeding [35]. Besides the observed association of fish consumption during weaning and the risk reduction of allergies [33,34], sea fish is a good source of iodine and, in case of fatty fish, of long-chain polyunsaturated fatty acids [36]. However, 40% of fish products in our survey were prepared with a low-fat species (pollack).

Nevertheless, there are several snack products on the German CCF market, a product category that is not necessary for a healthy diet during infancy.

Pouches were not listed in the 2000 market survey in Germany [28]. In the current survey, these products made up the largest group in the snack category. Pouches are criticized for their ingredients and texture [37] and are suspected to promote caries [38]. In Australia [37], pouches were mainly declared to older children aged 6 months on up. However, during this age, infants should be gradually accustomed to lumpy or solid foods [39,40].

Beverages are recommended to be offered when infants consume all three complementary meals [15]. Just as for older children, water is a suitable drink for infants. The American Academy of Pediatrics recommended avoiding any fruit juice during the first year of life [41], as the sugar content of fruit juices is similar to soft drinks. However, several products identified in this survey contained substantial amounts of sugar.

### 4.2. Energy and Nutrient Content

It is noticeable that CCF products, with the exception of meat products, provide more than 50% of calories from carbohydrates. The European directive on processed cereal-based foods and baby foods for infants and young children prescribes a low maximum fat content (6 g/100 kcal = 54% of energy for meat or cheese products, 4.5 g/100 kcal = 40.5% for all other products). These values were not reached by the majority of the products.

According to the directive, sodium salts may only be added to processed cereal-based foods for technological purposes and may not be added to products based on fruit or to desserts and puddings. Nevertheless, we found dietary salt as ingredient in several menus.

International authorities are unanimous in recommending that complementary foods should not be prepared with added sugar [7,9,12,14]. The current European directive only considers classical sweetener (i.e., sucrose, fructose, glucose, glucose syrups, and honey) and set maximum values for carbohydrates from these sources only for products including these ingredients [19]. The use of these classic sweeteners as sugar and honey was low in this survey. However, when fruit juice was included in the added sugars’ definition [31], the proportion of sweetened CCF clearly increased. Furthermore, the intense pureeing process liberates intrinsic sugar from fruits and vegetables. Hence, sugars from these foods can be considered free sugars too [31]. That is why a large portion of the total sugar as declared on CCF in our survey would have to be considered free sugars. It is noteworthy that the total sugar content for nearly all product categories was above 10%E, the free sugar limit recommended by the WHO for over 2–year–olds [30]. Therefore, there is a concern that such sweet CCF may contribute to sweet taste preference development, thus leading to excess energy intake [31]. This is particularly relevant against the background of a high prevalence of overweight and obesity even among preschool children (girls: 10.8%; boys: 7.3%) [42]. Hence, closing regulatory gaps to reduce sugar content in CCF is requested [31].

### 4.3. Fortification

The overall prevalence of fortification decreased from 70% in 1998 [28] to around 30% of products in the current survey. This could be due to a shift to a more organic produced CCF, which is not supposed to be fortified.

Vitamin B1, calcium, and vitamin D were the nutrients most commonly used for fortification. The extent to which this is necessary is questionable, since the reference values for calcium and vitamin B1 can be achieved without fortification [14]. However, the EU law prescribes that the amount of thiamin in processed cereal-based foods shall not be less than 100 μg/100 kcal. This high value requires fortification.

As vitamin D is supposed to be generally supplemented in the first year of life [9,43], the need for vitamin-D-fortified CCF is also questionable.

In the case of iron, young children are at special risk of iron deficiency, and iron-rich complementary foods are recommended. However, high iron intakes may have adverse effects [32]. Furthermore, to the best of our knowledge, there are no studies that prove the benefit of additional supplementation of meat-containing CCF, which is rich in heme iron with high bioavailability [14].

Iodine is a potential critical nutrient during weaning [12,28,44]. Except fish, nonfortified complementary food in general is low in iodine whether self-prepared or commercial [44]. Iodine fortification could be a strategy to improve iodine supply in infancy; however, risk assessments would be necessary to avoid excessive uptake. In our survey, less than 4% of all products were fortified with iodine. In a recent survey on cereal-based CCF, Theurich et al. reported *n* = 39 products fortified with iodine (24% of all products) [29]. This is in line with our results, as the highest iodine fortification prevalence was found in milk–cereal–meals. Theurich et al. [29] mentioned that these cereals reported follow-on formula in the ingredients list. According to the EU directive on infant and follow-on formula, an iodine content between 10 and 50 µg iodine/100 kcal is prescribed.

### 4.4. Ingredients

Our survey confirms the low variety of ingredients and tastes in CCF [18,21]. Particularly, bitter-tasting types, such as spinach, broccoli, and cauliflower, were used in a few products. The innate dislike of bitter-tasting substances in humans [45] can be overcome by repeated exposure [46]. Therefore, the complementary feeding period is regarded as a “window of opportunity” [47], when exposure to a wider range of flavors increases acceptance and reduces reluctance towards disliked and novel tastes even in the long term [48]. By the observed monotony of the types of fruits and vegetables in CCF, the chance to shape preferences in the sense of a healthy diet is missed. However, it should not be concealed that the variety of vegetables offered has increased in Germany in recent years. A 2012 review of fruits and vegetables in complementary foods identified 16 different vegetables in menus and vegetable preparations [18]. In the present market survey, there were 21 varieties, which is an increase of 31% since 2012.

The benefits of cereals during weaning are the impact on nutrient intake, in particular, iron; the promotion of an “adultlike” microbiota; and the semisolid texture and consistency [49]. Due to the higher iron and fiber intake, these benefits can be easier achieved by whole grain products instead of refined grains. In addition, the complementary feeding period is regarded as an important period for the acceptance of whole grains later in life [49].

It has not yet been systematically studied what influence the use of spices and flavors has on children’s long-term food preferences. Hence, to the best of our knowledge, there is no official recommendation on the use of spices or herbs during infancy.

### 4.5. Strengths and Limitations

We present data on products offered on the German food market. However, the food market is changing constantly. Furthermore, we did not have current consumption data of CCF among infants and toddlers, but it can be assumed that the market is regulated by demand, and only products are offered that are also bought by families. In addition, data on homemade complementary foods are lacking, so it is not possible to assess whether they are actually healthier. Older studies from Spain and Germany concluded that the differences in composition are not large [50,51], but the variety of vegetables is lower in CCF [18]. A further limitation is the use of declared energy and nutrient contents. No laboratory analyses were available, which could have provided more valid assessments of energy and nutrient contents. We decided to present nutrient contents per 100 g or as % of energy intake, as portion sizes vary with age. In the end, we must emphasize that our results and discussion apply only to children from affluent countries. In low-income countries, poor complementary feeding is a risk factor for malnutrition and may cause morbidity and mortality [52], and fortified complementary food may help to prevent nutrient deficiencies [53].

### 4.6. Conclusions

Both the categories of the products offered (e.g., snacks, caloric beverages) and the high sugar content of most products may promote unhealthy dietary habits during infancy and young childhood. Our market survey showed that sugar content was high in most product categories, even though manufacturers avoided adding sugar and other ingredients. As CCF consumption is common [20,27], parents need to be advised about the optimal selection of products. This includes, in particular, the avoidance of products with added salt and/or sweetened foods, including juice-sweetened products, snack foods, pouches, and desserts (and not using fruit products as desserts). Furthermore, parents should be encouraged to select a variety of vegetables that should not be mixed with fruit, as well as products with fish.

Furthermore, our market survey supports the claims of Hutchinson et al. [31], that is, the ban of added sugars and sweet snacks, the limited use of pureed fruit in some food categories, and the limitation of total sugar content of “savory” snacks. We would further support to ban the production of sugar-sweetened beverages intended for infants, even if the sugar is natural in the case of juices and mixtures of juice with tea or water.

## Figures and Tables

**Table 1:** Product categories and declared age of infants’ commercial complementary foods, results of a 2020 market survey in Germany.

Product Category	Total	After 4th Month/ from the Age of 5 Months	After 6th/7th Month	After 8th/9th Month	After 10th/12th Month
	*n* (%) ^1^	*n* (%) ^2^	*n* (%) ^2^	*n* (%) ^2^	*n* (%) ^2^
*Full meals (n = 328)*	Data	Data	Data	Data	Data
Menus	180 (21.7)	38 (21.1)	21 (11.7)	65 (36.1)	56 (31.1)
Milk–Cereal–Meals	85 (10.3)	25 (29.4)	41 (48.2)	10 (11.8)	9 (10.6)
Cereal–Fruit–Meals	63 (7.6)	19 (30.2)	28 (44.4)	11 (17.5)	5 (7.9)
*Meal components (n = 288)*					
Fruit	137 (16.5)	93 (78.9)	22 (16.1)	6 (4.4)	16 (11.7)
Cereals	68 (8.2)	38 (55.9)	20 (29.4)	4 (5.9)	6 (8.8)
Vegetables	63 (7.6)	53 (84.1)	10 (15.9)	-	-
Meat	12 (1.4)	10 (83.3)	2 (16.7)	-	-
Fish	2 (0.2)	1 (50)	1 (50)		
Oil	6 (0.7)	6 (100)	-	-	-
*Snacks (n = 159)*					
Pouches	111 (13.4)	12 (10.8)	78 (70.3)	14 (12.6)	7 (6.3)
Snack foods	43 (5.2)	-	8 (18.6)	32 (74.4)	3 (7.0)
Desserts	5 (0.6)	-	1 (20.0)	-	4 (80.0)
Beverages *(n = 54)*					
Tea, water, juices	54 (6.5)	45 (83.3)	6 (11.1)	1 (1.9)	2 (3.7)
*Total*	829 (100)	340 (41.0)	238 (28.7)	143 (17.3)	108 (13.0)

^1^ Percentage of all products. ^2^ Percentage of the respective product category.

**Table 2:** Declared energy and nutrient contents per 100 g of commercial complementary food products for infants and toddlers, results of a 2020 market survey in Germany (values are medians (quartile 1, quartile 3) ^1^.

Product Category (*n*)	Energy (kcal)	Protein (g)	Fat (g)	SFA (g)	Carbohydrates (g)	Total Sugar (g)	Salt (mg)
**Infants**							
*Full meals*							
Menus (*n* = 180)	66 (61;70)	2.5 (22.2; 2.8)	2.4 (1.9; 2.8)	0.5 (0.4; 0.7)	7.7 (7.0; 8.7)	2.1 (1.6; 2.5)	0.08 (0.05; 0.03)
Milk–cereal–meals (*n* = 85)	92 (76;103)	3.0 (2.6; 3.5)	2.5 (2.2; 2.9)	0.9 (0.7; 1.1)	14.0 (11.0; 16.1)	6.3 (4.8; 7.3)	0.08 (0.07; 0.09)
Fruit–cereal–meals (*n* = 63)	66 (60; 72)	1.0 (0.8; 1.1)	0.4 (0.3; 0.6)	0.1 (0.0; 0.2)	13.6 (11.9; 15.0)	9.6 (7.6; 10.8)	0.03 (0.01; 0.05)
*Meal components*							
Fruits (*n* = 137)	57 (52; 62)	0.5 (0.4; 0.7)	0.2 (0.1; 0.5)	0.0 (0.0; 0.2)	11.8 (11.1; 13.0)	10.1 (9.1; 10.8)	0.05 (0.01; 0.05)
Cereals ^2^ (*n* = 68)	376 (363; 388)	11.0 (9.3; 12.5)	3.2 (2.1; 4.5)	0.7 (0.5; 0.9)	70.0 (6.9; 78.1)	1.5 (0.8; 5.7)	0.01 (0.01; 0.03)
Vegetables (*n* = 63)	41 (31; 54)	0.9 (0.7; 1.2)	1.2 (0.2; 1.8)	0.1 (0.0; 0.2)	6.5 (5.0; 7.5)	2.6 (2.0; 3.8)	0.05 (0.02; 0.08)
Meat (*n* = 12)	102 (90; 108)	8.1 (7.8; 8.9)	4.6 (3.1; 5.8)	1.7 (1.0; 2.1)	6.0 (4.7; 6.4)	0.0 (0.0; 0.2)	0.07 (0.04; 0.08)
*Snacks and beverages*							
Pouches (*n* = 111)	63 (54; 72)	0.8 (0.6; 1.1)	0.5 (0.3; 0.6)	0.1 (0.1; 0.2)	12.0 (11.0; 14.0)	9.9 (8.1; 12.0)	0.01 (0.01; 0.05)
Snack foods (*n* = 43)	390 (370; 429)	8.0 (6.9; 10.0)	4.4 (0.9; 11.0)	0.8 (0.2; 1.5)	76.0 (70.0; 82.0)	7.5 (2.7; 13.0)	0.03 (0.01; 0.05)
Desserts (*n* = 5)	84 (82; 84)	3.5 (3.0; 3.6)	2.7 (1.7; 3.1)	1.2 (1.1; 1.7)	12.4 (11.8; 13.1)	8.4 (8.4; 8.6)	0.13 (0.10; 0.13)
Beverages ^3^ (*n* = 44)	30 (20; 47)	0.2 (0.0; 0.5)	0.1 (0.0; 0.2)	0.0 (0.0; 0.0)	6.5 (4.8; 10.9)	6.2 (4.4; 9.8)	0.05 (0.01; 0.05)
Toddler							
Menus (*n* = 40)	70 (67; 75)	2.6 (2.5; 2.9)	2.4 (2.3; 2.6)	0.5 (0.4; 0.8)	8.8 (8.2; 9.8)	1.6 (1.4; 1.9)	0.43 (0.36; 0.48)
Cereals (*n* = 12)	370 (35; 377)	11.1 (10.5; 12.2)	5.6 (3.8; 5.9)	1.0 (0.7; 1.2)	63.1 (90.7; 67.7)	8.5 (6.3; 10.8)	0.01 (0.01; 0.05)
Pouches (*n* = 60)	59 (55; 64)	0.6 (0.4; 0.8)	0.4 (0.2; 0.5)	0.0 (0.0; 0.2)	12.0 (11.3; 12.9)	10.8 (10.1; 11.5)	0.05 (0.01; 0.05)
Snack foods (*n* = 77)	383 (366; 416)	5.7 (4.4; 7.6)	8.6 (6.6; 13.7)	2.0 (0.9; 3.6)	69.0 (64.8; 72.0)	28.8 (12.1; 41.0)	0.05 (0.03; 0.15)
Desserts (*n* = 6)	84 (82; 90)	3.2 (3.1; 3.6)	2.3 (1.7; 3.3)	1.7 (1.2; 2.2)	12.4 (11.7; 13.3)	8.4 (7.4; 8.6)	0.12 (0.08; 0.15)
Beverages ^3^ (*n* = 25)	18 (16; 21)	0.0 (0.0; 0.1)	0.1 (0.0; 0.1)	0.0 (0.0; 0.0)	3.8 (3.5; 4.5)	3.5 (3.1; 4.2)	0.05 (0.05; 0.05)

^1^ Due to the small number of products, the product categories oil and fish were not shown; SFA saturated fatty acids. ^2^ Content of the dry product. ^3^ Ready–to–drink beverages only, excluding instant teas and bagged teas, missing values for salt content in beverages: *n* = 3 (infant products) and *n*= 6 (toddler products).

**Table 3:** Nutrient fortification ^1^ in commercial complementary foods (CCF), results of a 2020 market survey in Germany ^2^.

Product Category (*n*)	Total *n* (%)	Vitamin B1 *n* (%)	Vitamin C *n* (%)	Iron *n* (%)	Calcium *n* (%)	Vitamin D *n* (%)	Iodine *n* (%)
*Infants (829)*	264 (31.9)	185 (22.3)	91 (11.0)	63 (7.6)	61 (7.4)	59 (7.1)	32 (3.9)
Menus (180)	22 (12.2)	-	-	22 (12.2)	-	-	-
Milk–cereal–meal (85)	79 (92.9)	77 (90.6)	33 (38.8)	36 (42.4)	57 (67.1)	57 (67.1)	32 (37.7)
Cereal fruit meal (63)	11 (17.5)	7 (11.1)	5 (7.9)	-	-	-	-
Fruits (137)	25 (18.3)	4 (2.9)	22 (16.1)	-	4 (2.9)	-	-
Cereals (68)	68 (100.0)	68 (100.0)	1 (1.5)	1 (1.5)	-	1 (1.5)	-
Vegetables	2 (1.8)	-	1 (1.6)	2 (3.2)	-	-	-
Pouches	2 (1.8)	-	2 (1.8)				
Snack foods (43)	28 (67.4)	28 (65.1)	-	-	-	-	-
Desserts	1 (20%)	1 (20.0)	1	-	-	1 (20.0)	-
Beverages (54)	26 (48.2)	-	26 (48.2)	2 (3.7)	-	-	-
*Toddlers (228)*	53 (23.3)	30 (13.2)	16 (7.0)	9 (4.0)	2 (0.9)	-	-
Menus (23)	3 (13.0)	-	-	3 (13.0)	-	-	-
Cereals (12)	11 (91.7)	11 (91.7)	-	-	-	-	-
Snack foods (77)	21 (27.3)	18 (23.3)	-	4 (5.2)	1 (1.3	-	-
Pouches (60)	15 (25%)	-	15 (25)	2 (3.3)	-	-	-
Beverages (26)	2 (7.7)	-	1 (3.9)	-	1 (3.9)	-	-
Pasta (2)	1 (14.3)	1 (14.3)	-	-	-	-	-
*Total (1057)*	317 (30.0)	215 (20.3)	107 (10.1)	72 (6.8)	63 (6.0)	59 (5.6)	32 (3.0)

^1^ Fortification with vitamin B2, vitamin B6, vitamin B12, vitamin K, niacin, biotin, zinc, pantothenic acid: <1% of products, fortification with vitamin E, folate < 3% of products; no fortified products found among oils, meat, and fish. ^2^ Percentage of fortified products in this category.

## Data Availability

The datasets used and/or analyzed during the current study are available from the corresponding author on reasonable request.
